# Consequences of traffic noise in residents of Karachi, Pakistan

**DOI:** 10.12669/pjms.312.6367

**Published:** 2015

**Authors:** Imtiaz Ather Siddiqui, Sohaib Nizami, Rida Rouf Chandio, Saad Nizami, Nazish Sikander, Sana Ashraf

**Affiliations:** 1 Dr. Imtiaz Ather Siddiqui, DLO, Department of Ear, Nose, Throat, Head & Neck Surgery, Jinnah Postgraduate Medical Center, Karachi, Pakistan and Jinnah Sindh Medical University, Karachi, Pakistan; 2 Sohaib Nizami, Final Year MBBS student, Department of Ear, Nose, Throat, Head & Neck Surgery, Jinnah Postgraduate Medical Center, Karachi, Pakistan and Jinnah Sindh Medical University, Karachi, Pakistan; 3 Rida Rouf Chandio, Final Year MBBS student, Department of Ear, Nose, Throat, Head & Neck Surgery, Jinnah Postgraduate Medical Center, Karachi, Pakistan and Jinnah Sindh Medical University, Karachi, Pakistan; 4 Saad Nizami, Final Year MBBS student, Department of Ear, Nose, Throat, Head & Neck Surgery, Jinnah Postgraduate Medical Center, Karachi, Pakistan and Jinnah Sindh Medical University, Karachi, Pakistan; 5 Nazish Sikander, Final Year MBBS student, Department of Ear, Nose, Throat, Head & Neck Surgery, Jinnah Postgraduate Medical Center, Karachi, Pakistan and Jinnah Sindh Medical University, Karachi, Pakistan; 6 Sana Ashraf, Final Year MBBS student, Department of Ear, Nose, Throat, Head & Neck Surgery, Jinnah Postgraduate Medical Center, Karachi, Pakistan and Jinnah Sindh Medical University, Karachi, Pakistan

**Keywords:** Noise, Traffic, Hearing impairment, Noise Pollution, Noise induced hearing loss, Tinnitus

## Abstract

**Objective:**

To find out effect of road traffic noise on human beings in busy places of Karachi, working at these places and to compare its results with the previously done studies on this subject.

**Methods:**

This prospective epidemiological study was designed to evaluate effects of Noise induced hearing Loss due to road traffic at different places (Gurumander, Tibet Centre, Marry Weather Tower) of Karachi. A sample of 125 cases were randomly selected who had noise exposure of 90 dB or above of their surroundings for more than 6 months. The study was conducted from October 1^st^ 2013 to January 1^st^ 2013.

**Results:**

The minimum age was 18 years while maximum age was 47 years. The age group found most affected was from 23 years to 27 years. The males were 84% and females 16%. Subjects exposed to noise for more than 12 hours per day were 36.8%. Varying degree of hearing loss was evaluated in subjects where 17.6% were normal, 33.6% had mild hearing loss, 45.6% had moderate and 3.2% had moderately severe hearing loss. Traffic noise was found to bother 55.2% of subjects.

**Conclusion:**

Analysis of data indicates an enormous increase in noise levels as compared to previous studies. This study establishes that there exists a concrete direct link between NIHL and duration of exposure to noise above permissible levels. Traffic authorities should initiate measures to reduce the noise levels in the city particularly at more noisy places.

## INTRODUCTION

Noise was born the day wheel was invented. In fact the invention of wheel is the birth of technology and noise both. Noise is any unjustifiable interference within the normal human hearing frequency range that is from 20 Hz to 20000 Hz (NIOSH, 1991).^1^

Rapid urbanization in the past many years has led to increase in noise pollution. It has adverse effects not only on hearing but has many psychological and pathological impacts on human health. Noise is a part of daily human activity and is thus unavoidable. Apparently one cannot distinguish between noise and sound.

Noise is any kind of sound that is perceived as unharmonious or disorganized. It is broadly categorized into occupational noise and environmental noise. Environmental noise is one originating from various activities except noise produced at industrial workplace i.e. occupational noise. The major contributors to the environmental noise are road traffic, rail traffic and air traffic while the major contributors of occupational noise include industrial machinery, ventilation system, construction equipment and workplace noise along with industrial noise. Vulnerable groups within the population which are usually encountered are factory workers, labourers, auto rickshaw drivers, shopkeepers, street vendors, mechanics, denters and traffic Police personal.^2^ Exposure to noise in day to day life leads to mood changes, anxiety, sleep disturbance and reduced work efficiency. Ceremonial noise in residential area is also a source of discomfort and agitation. NIHL may even cause platelet clumping which may lead to cardiovascular ailments.^3^

Prolonged exposure to noise can cause noise-induced hearing loss which is unknowingly harmful and leads to irreversible hearing impairment.^4^ The people who are chronically exposed to noise are more prone towards developing reduced hearing sensitivity as compared to the non-exposed people. Industrial noise in Pakistan is greatest emerging from weaving looms of textile industry, steel mills and airports in the largest cities. For instance, average noise levels in a textile mill in Karachi were found to vary between 85 and 112 dB. In the sheet metal industry, 8% of workers had noise-induced hearing impairment. Another study of Karachi textile workers found that 22% of those exposed to noise had noise-induced hearing loss compared to 2% of controls. More than half of the cases with noise induced hearing loss also had tinnitus.^2^ A significant difference in prevalence of hearing loss (more than 30db) between the noise exposed and non noise exposed group (P <0.5) in aviation workers was noted in a study conducted in Karachi.^4^

The permissible noise exposure varies from country to country. Occupational safety and Health Administration Agency (OSHA) in US allows exposure of up to 90 dB for a period of 8hours/day, whereas most other countries allow 85 dB for 8 hours duration.^5^ In Europe noise-induced hearing loss was estimated for 10.3% of all occupational diseases between 1999 and 2001. In Washington, USA, a survey was conducted in (2009) which had analyzed 1999–2004 data from the National Health and Nutrition Examination Survey (NHANES) to determine the burden of occupational exposure from self-report. They found out that 22 million workers (17% of the population-weighted survey) reported exposure to hazardous occupational noise and out of these, 34% reported nonuse of hearing protective devices (HPDs).^6^

The prevalence of NIHL in New Zealand as per ACC data reveals that there has been a substantial increase in the number of new NIHL claims annually, rising from 2823 in July 1995-June 1996, to 5580 in July 2005-June 2006.^7^ In 2003 the relevant medical costs(hearing aids, treatment and assessment) were as high as five times the cost in 1995.^8^ In Brazil, the presence of suspected NIHL was 28.5% and the finding was higher among those working in the noisier areas than those working in lower noise areas (38.8% versus 24.2%).^9^ In India another study emphasized on the traffic policemen working for 10–12 hours daily in a noisy surroundings. 84% of the sample showed hearing loss and reported at least some hindrance in hearing by one or both ears.^10^

It has been officially recorded that Karachi is ranked eighth in world figures for noise pollution.^11^ According to WHO, responsibility lies on the government to protect the community from exposure to unwanted noise and reduce noise emission and not just the sources and take necessary measures to protect risk groups.^2^

This study was planned to find out effect of road traffic noise on human livings and working at these places for protection of NIHL. The study was conducted in such a way that effects of noise on residents and workers of these noisy places were also determined. Another aim in starting this study was to compare its results with the previously done studies on this topic.

## METHODS

A hospital based prospective epidemiological study was conducted at Jinnah Sindh Medical University (JSMU) designed to evaluate effects of noise-induced hearing loss due to road traffic at Gurumander, Tibet Center, Marry Weather Tower places of Karachi. A sample of 125 cases were randomly selected who had noise exposure of 90 db or above of their surroundings for more than 6 months.

Noise levels at different busy traffic intersections of Karachi were taken by a sound level meter branded “Standard Sound Level Meter ST-85A”. The noise meter was calibrated as per specifications laid by American Audiological Academy. Five spots were selected where noise level was recorded between 100db to 110db from dawn to dusk. Subjects were selected who were workers of different trades at these places.

Data was collected on a specified Performa focused on subject’s profession, duration of noise exposure at work place, presence of tinnitus and other symptoms of NIHL and community response to noise.

Consent was taken from each subject and pure tone audiometery was carried out in a sound proof room at ENT Department, Jinnah Postgraduate Medical Centre. Pure Tone Audiogram on a screening mode of 500Hz, 1000Hz, 2000Hz, 4000Hz and 8000Hz was obtained in a sound proof room. The biase were eliminated to significant degree during the study. The data was analyzed on IBM SPSS Statistics v20. The duration of study was from 1^st^ October 2012 to 1^st^ January 2013. 

### Exclusion Criteria

This consisted of:

- Age above 50 years and below 10 years.
- Any history of previous Otological disease.
- History of Ototoxic drug intake for a period of more than a month.
- History of high-grade fever for more than one week.
- Known case of Diabetes Mellitus, Hypertension or any systemic organic disease.

## RESULTS

In a series of 125 subjects, the minimum age was 18 years while maximum age was 47 years. The mean age was calculated to be 30.87 years. The age group found most affected was from 23 years to 27 years.

**Fig.1:**
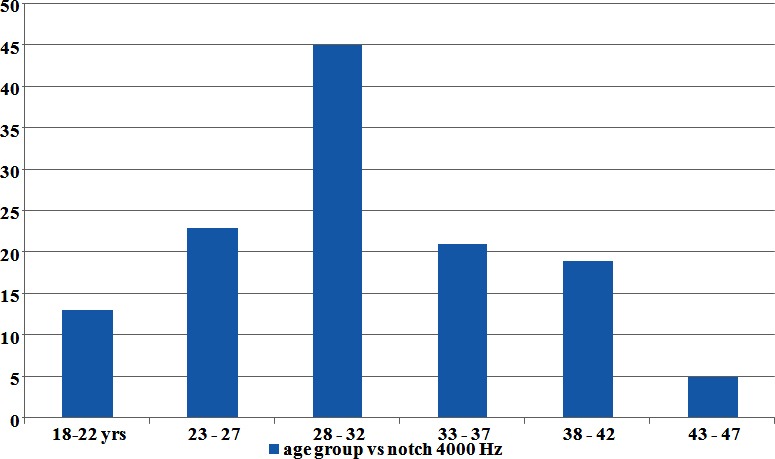
Age group having notch at 4000Hz.

The males were 84% (105) and females 16% (20). Male- to female ratio came 5.25:1. Subjects exposed to noise for more than 12 hours per day were 36.8% (46). Subjects who complained of tinnitus were 32.8% (41). A notch at 4000 Hz on PTA air conduction was found in 80% (100) Fig.1. This notch is pathognomonic of NIHL whereas only 12.8% (16) were aware of hearing impairment.

Varying degree of hearing loss was evaluated in subjects where 17.6% (22) were normal, 33.6% (42) had mild hearing loss, 45.6% (57) had moderate and 3.2% (4) had moderately severe (Table-I).

**Table-I:** Degree of hearing loss encountered in subjects.

Hearing loss (Decibel)	Degree of hearing loss	No. of subjects
<26	Normal hearing	17.6 %
26-40	Mild	33.6 %
41-45	Moderate	45.6 %
56-70	Moderately Severe	3.2 %
71-90	Severe	0
>90	Profound	0

**Fig.2:**
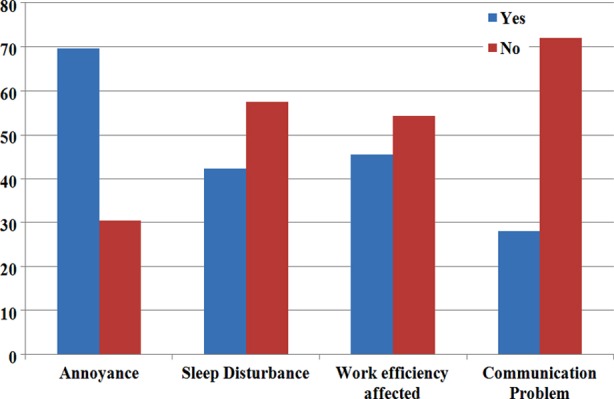
Community response of society to noise.

Subjects experiencing hearing impairment for loud conversation were 4% (5), for telephone bell 10.4% (13) and television 23.2% (29). Community response to noise was also assessed and 69.6% (87) experienced annoyance due to noise, 42.4% (53) suffered sleep disturbance and 45.6% (57) claimed their working capacity being compromised due to noise (Fig.2). Moreover, communication difficulties were encountered by 28% (35) and 13.6% (17) had vertigo. Traffic noise was found to bother 55.2% (69) of subjects.

## DISCUSSION

Excessive noise is a health hazard across the globe with considerable social and physiological impacts including NIHL. Environmental noise among all categories of noise affects a bigger population than other categories. Road traffic noise has been established as the most significant source in the city of Karachi.^11^ The population of Karachi remains unaware of this disability until irreversible damage has occurred. A substantial amount of Pakistan’s population living in the mega metropolitan city of Karachi is at risk of hearing loss or this may lead to an overall high incidence of hearing impairment if drastic preventive measures are not taken.

This study was on traffic noise which affects adversely, unknowingly to all age groups of population. There are other studies carried out in Karachi such as study done by Itrat Javed et al.^12^ This study encompasses almost most of our objectives but the gender is not mentioned which is presumably all subjects are males while our study has 84% males and 16% females. This represents more close analysis. This study is silent on the type, calibration of the Pure Tone Audiometer and environment of the recording. We have mentioned the type and adopted the American standards of calibration in our study. In Itrat Javed’s study,^12^ the subjects belong to three categories of noise affectees were selected i.e. Rickshaw drivers (13.5%), traffic constables (39.5%) and shopkeepers (29.5%). In our study there were eight categories comprising from all walks of life that are at a potential danger of hearing impairment due to road traffic noise. They are labourer working across the road (46.03%), auto rickshaw drivers (17.46%), shopkeepers (13.49), auto workshop mechanic (9.52%) traffic constables (5.58%) street venders (4.76%) and auto workshop denters (3.16%).

Another study on Spatial and Temporal patterns of noise exposure due to road traffic in Karachi^13^ found that maximum peak noise was over 101 dB which is close to 110dB, this is the level which can cause possible hearing impairment as per WHO guide lines. In our study residents and workers of above mentioned places of Karachi has developed hearing impairment due to noise exposure. This needs attention of authorities to reduce noise level for prevention of hearing impairment and annoyance.

Earliest study on road traffic noise in Karachi was conducted by Zaidi S.H in 1989. Which noise levels were measured at similar locations of Karachi as in our study. In this study 99dB was the noise level while this has increased hence people suffering from hearing impairment have also increased in our study. Road Traffic was also identified as source of noise. Our study has validated this old finding as it is still there and needs attention for authorities to prevent hearing impairment.^14^

Keeping in view the sheer number of persons exposed to noise, probed in depth the relationship between environmental noise and its effect on health. Madrid (Spain) is a densely populated metropolitan city like Karachi. Here 80% of all environmental noise is attributed to road traffic. This is an identical situation to our study. The study carried out to quantify avoidable deaths due to impact of noise level on daily cardiovascular and respiratory mortality among age group>65 years in Madrid.^15^ Our study shows an enormous increase in noise level in Karachi as compared to previous studies. This in turn increases the impact on cardiovascular and respiratory systems of Karachi citizens.

Traffic noise exposure being the dominant community, with annoyance, which is an indicator of chronic health conditions. A study was also conducted on adult Indian population to explore association between residential road traffic noise and self-reported annoyance. This cross sectional study revealed the prevalence of annoyance was more for males. The vulnerable age groups were 34-40 years followed by 50-60 years. It also found association of residential road traffic noise with annoyance.^16^

In contrast our study noise effected sub age group was different i.e. 28-32 followed by 23-27 years, indicating younger population in contrast to Indian study. While annoyance is almost reaching 70% in our study.

## CONCLUSION

There is an enormous increase in noise levels in Karachi as compared to previous studies. This study establishes that there exists a concrete direct link between NIHL and duration of exposure to noise above permissible levels. Traffic authorities should initiate measures to reduce the noise levels in the city particularly at high noisy places. Noise meters should be installed on major noise producing intersections. When these noise meters indicate noise levels above 90 dB (permissible level) traffic should be diverted to other areas to reduce noise level.
